# Mechanisms by which carbamoylated high-density lipoprotein (C-HDL) promotes calcific aortic valve disease and exploration of potential targeted therapies

**DOI:** 10.1515/biol-2025-1367

**Published:** 2026-09-08

**Authors:** Min Han, Wang Jiang, Yalong Yang, Ran Chen

**Affiliations:** Department of Cardiovascular Diseases, The Affiliated ChangshaCentral Hospital, Hengyang Medical School, University of South China, 161 Shaoshan Road, Changsha, HuNan, China; Department of Gastroenterology, Jiangxi Provincial Key Laboratory of Digestive Diseases, Jiangxi Clinical Research Center for Gastroenterology, Digestive Disease Hospital, The First Affiliated Hospital, Jiangxi Medical College, Nanchang University, Nanchang, Jiangxi, China

**Keywords:** carbamoylated high-density lipoprotein, calcific aortic valve disease, bioinformatics

## Abstract

Calcific aortic valve disease (CAVD) is a progressive fibrocalcific illness for which no effective pharmaceutical treatment exists. This study investigated whether carbamoylated high-density lipoprotein (C-HDL), a defective type of HDL that can develop during inflammation, contributes to CAVD progression and the involved molecular pathways. Male ApoE^−/−^ mice were divided into three groups: CAVD model, cyanate-treated, and inhibitor, and analyzed after 12 weeks. C57BL/6 mice on a regular diet served as blank controls. Serum paraoxonase-1 (PON1), aortic valve calcification, cluster of differentiation 31 (CD31), phosphorylated nuclear factor kappa B p65 (p-p65), NOTCH receptor 1 (NOTCH1), and runt-related transcription factor 2 (RUNX2) were evaluated. In parallel, using RNA sequencing (RNA-seq), Gene Ontology (GO) and Kyoto Encyclopedia of Genes and Genomes (KEGG) enrichment analyses, protein-protein interaction (PPI) network analysis, and quantitative real-time polymerase chain reaction. Cyanate treatment reduced serum PON1 levels, increased von Kossa-positive calcium deposition, and raised CD31, p-p65, NOTCH1, and RUNX2 levels compared with the model group, but Gly partially corrected these effects. Transcriptomic research identified 270 C-HDL-associated differentially expressed genes (DEGs) enriched in pathways associated with inflammatory signaling and NF-κB activity. Five potential hub genes (BIRC6, PIK3R1, ATM, IFIH1, and DDX58) were discovered and verified using qRT-PCR. These data show that C-HDL may accelerate CAVD by disrupting valve endothelial homeostasis and stimulating inflammatory signaling, and they identify potential molecular targets for future functional validation.

## 1 Introduction

Calcific aortic valve disease (CAVD) is a degenerative valvular illness characterized by leaflet thickness and fibrosis, which eventually results in calcium deposition and reduced valve motion. Clinically, it can range from asymptomatic aortic valve sclerosis to severe aortic stenosis, heart failure, and sudden death. Although surgical and transcatheter aortic valve replacement are beneficial for advanced disease, no pharmacological therapy has been shown to slow early or intermediate-stage CAVD progression [1]. Identifying upstream genetic causes and modifiable treatment targets is a top clinical and translational objective.

The degeneration process of CAVD is not passive. An active disease model with lipid accumulation, inflammation, oxidative stress, endothelial dysfunction, immune-cell infiltration, extracellular matrix remodeling, and osteogenic differentiation of valve interstitial cells is supported by current data [2], 3]. While nuclear factor of kappa light chain enhancer of B cells (NF-κB) activation encourages inflammatory amplification and osteogenic gene expression, Runt-related transcription factor 2 (RUNX2) is a key regulator of osteogenic programming [4].

Valve development and endothelial-mesenchymal balance are also influenced by NOTCH receptor 1 (NOTCH1), and valvular calcification has been connected to disruptions in NOTCH signaling [5]. Therefore, understanding the course of CAVD requires a molecular framework that links defective lipoproteins, endothelial damage, inflammatory signaling, and osteogenic differentiation.

High-density lipoprotein (HDL) facilitates reverse cholesterol transfer and has antioxidant, anti-inflammatory, and endothelial-protective characteristics. However, HDL function, such as cholesterol efflux capacity and antioxidant activity, may provide more biological information than HDL cholesterol content alone [6], 7]. Under chronic inflammation, metabolic stress, and chronic kidney disease, HDL can change in protein composition and post-translational modification, resulting in diminished protective activity and, in some cases, a shift toward a pro-inflammatory phenotype [8], 9]. In calcific aortic stenosis, reduced HDL cholesterol export capacity has been linked to disease severity, suggesting that HDL dysfunction may play a role in valve pathology [10].

Carbamylation is a post-translational modification in which isocyanic acid interacts with lysine residues to produce homocitrulline, changing protein structure and function. Isocyanic acid can be produced through urea breakdown or myeloperoxidase-mediated thiocyanate oxidation, especially in inflammatory or uremic conditions. Carbamylated lipoproteins have been linked to vascular inflammation, endothelial dysfunction, monocyte adhesion, and accelerated atherogenic processes [11]. Carbamoylated HDL (C-HDL) may thus establish a biologically plausible link between systemic inflammation, HDL malfunction, valvular endothelial damage, and the evolution of CAVD.

In this study, we established a model related to CAVD using ApoE−/− mice fed a high-fat diet, and we employed drinking water containing cyanate to augment the *in vivo* carbamylation burden. In order to determine whether attenuating cyanate-associated alterations may lessen the biochemical, histological, endothelial, and molecular characteristics of valve calcification, glycylglycine (Gly) was employed as an inhibitor. Additionally, we investigated whether C-HDL directly modifies valve-cell transcriptional programs using human aortic valve interstitial cells (AVICs).

Key regulatory nodes may not be captured by candidate-gene techniques alone because CAVD development involves numerous interacting pathways. The characterization of autophagy, immunological infiltration, pyroptosis, and hub-gene signatures in CAVD has increasingly been done using bioinformatics techniques [12], [13], [14], [15]. In order to find C-HDL-associated transcriptional alterations and potential hub genes in AVICs, we combined RNA sequencing (RNA-seq), Gene Ontology (GO), Kyoto Encyclopedia of Genes and Genomes (KEGG) enrichment analyses, protein-protein interaction (PPI) network construction, and quantitative real-time polymerase chain reaction (qRT-PCR) validation.

## 2 Materials

### 2.1 Laboratory animals

Jiangsu Jicui Yaokang Biotechnology Co., Ltd. provided 18 male ApoE−/− mice aged 7–8 weeks (license No. SCXK [Su] 2023-0009) that were clear of pathogens. ApoE−/− mice were allocated evenly into three groups: model, cyanate, and inhibitor. Six male specific-pathogen-free C57BL/6 mice of the same age range were obtained from the same supplier and served as blank controls.

**Ethical approval:** The research related to animal use has been complied with all the relevant national regulations and institutional policies for the care and use of animals, and has been approved by the Laboratory Animal Ethics Committee, Jiangxi Zhonghong Boyuan Biotechnology Co., Ltd.(Certificate No.LL202407010002).

### 2.2 Cell culture

Human AVICs were used to establish an *in vitro* CAVD-related model and to determine whether C-HDL directly alters the transcriptional profile of valve cells. Cells were maintained in high-glucose Dulbecco’s modified Eagle medium at 37 °C in 5 % CO_2_ and were randomly assigned to PBS control or C-HDL treatment (75 μg/mL). This cell model was used for RNA-seq, bioinformatics screening, and qRT-PCR validation of candidate hub genes.

## 3 Methods

### 3.1 Animal grouping and housing

Eighteen Male ApoE−/− mice were randomly assigned to the model, cyanate, and inhibition groups. While six male C57BL/6 mice were used to be control group. Before intervention, the animals got used to it for seven days at typical temperatures of 25 °C and 40 % humidity.

### 3.2 Establishment of the experimental CAVD mouse model

In order to trigger CAVD-related modifications, ApoE−/− mice in the model-related groups were fed a high-fat diet for 12 weeks following acclimatization. Blank controls were C57BL/6 mice provided a regular diet. Five times a week, measurements were made of body weight, appearance, activity, food intake, and mortality. The authorized animal ethics protocol was followed for managing animals who met predetermined humane objectives, such as significant weight loss or significantly decreased activity.

### 3.3 Establishment of a C-HDL-modified mouse model

To increase the *in vivo* carbamylation burden, mice in the cyanate and cyanate plus Gly groups were given cyanate-containing drinking water (1 mg/L) in addition to their high-fat meal. Drinking bottles were replaced every three days, and water use was tracked. Throughout the intervention period, mice in the inhibitor group received 500 mg/kg of Gly intraperitoneally at 10:00 a.m. Gly was utilized as a carbamylation-related inhibitory intervention to see if suppressing cyanate-induced alterations could diminish valve calcification and molecular activation.

### 3.4 Von Kossa staining

Aortic root tissues containing valve leaflets were fixed, embedded in paraffin, sectioned, and stained using a standard von Kossa protocol. Calcified regions were identified as dark brown-to-black deposits. Three consecutive sections from the same anatomical level of each animal were photographed and analyzed.

### 3.5 Serum enzyme-linked immunosorbent assay

Serum PON1 concentrations were measured using a commercial enzyme-linked immunosorbent assay kit according to the manufacturer’s instructions. Absorbance was measured at 450 nm, and concentrations were calculated from the standard curve.

### 3.6 Immunohistochemical staining

Paraffin-embedded valve sections (4 μm) were deparaffinized, rehydrated, subjected to citrate-buffer antigen retrieval (pH 6.0), blocked with 3 % BSA, and incubated overnight at 4 °C with anti-CD31 primary antibody (1:200). After incubation with secondary antibody, sections were developed, counterstained, dehydrated, and mounted. CD31-positive area was quantified from comparable valve regions using ImageJ.

### 3.7 Western blotting

Valve and annular tissues stored at −80 °C were lysed in radioimmunoprecipitation assay buffer containing phosphatase inhibitors. Protein concentrations were determined using a bicinchoninic acid assay. Equal amounts of protein were separated by sodium dodecyl sulfate-polyacrylamide gel electrophoresis, transferred to membranes, blocked, and incubated overnight at 4 °C with primary antibodies against p-p65, NOTCH1, RUNX2, and glyceraldehyde-3-phosphate dehydrogenase (GAPDH). After incubation with secondary antibody, bands were detected and quantified by densitometry, with GAPDH used as the loading control.

### 3.8 Transcriptome sequencing of AVICs

Human AVICs were treated with C-HDL (75 μg/mL) or PBS for 8 h and harvested for transcriptome sequencing. Total RNA was extracted with TRIzol reagent. RNA concentration and purity were assessed using a NanoDrop 2000 spectrophotometer (A260/A280 ratio, 1.9–2.1), and RNA integrity was assessed using an Agilent 2100 Bioanalyzer (RNA integrity number ≥7.0). Libraries were sequenced on the Illumina NovaSeq 6000 platform using paired-end 150-bp reads, with at least 20 million reads per sample.

### 3.9 Differential expression analysis

RNA-seq data were processed in R. Expression profiles were normalized before differential expression analysis. Linear modeling and empirical Bayes statistics were applied to compare the C-HDL and PBS control groups. DEGs were defined as genes with adjusted *P* < 0.05 and an absolute log2 fold change ≥1. Volcano plots and distribution plots were generated for data visualization.

### 3.10 GO and KEGG enrichment analyses

GO and KEGG enrichment analyses were performed to identify biological processes, cellular components, molecular functions, and pathways associated with C-HDL-related DEGs. Enrichment was considered significant at *P* < 0.05 and false discovery rate <0.10. Results were visualized using bar plots, bubble plots, and network diagrams.

### 3.11 PPI network construction and hub-gene identification

PPI networks were constructed using STRING with *Homo sapiens* as the reference species and a combined score ≥0.4 as the interaction threshold. The resulting network was imported into Cytoscape for visualization. Molecular Complex Detection was used to identify densely connected modules, and candidate hub genes were prioritized according to node degree (degree ≥15) and biological relevance to inflammation, stress signaling, cell survival, and valve calcification.

### 3.12 RNA extraction and qRT-PCR

Total RNA from AVICs was reverse-transcribed using a commercial reverse-transcription kit according to the manufacturer’s instructions. Relative mRNA expression was quantified by qRT-PCR and calculated using the 2−ΔΔCt method with GAPDH as the internal reference. Primer sequences are listed in Table 1. Graphs were generated using GraphPad Prism 8.0.2.

**Table 1:** Primer sequences used for qRT-PCR.

Primer name	Primer sequence (5′→3′)
GAPDH forward	GTC​TCC​TCT​GAC​TTC​AAC​AGC​G
GAPDH reverse	ACC​ACC​CTG​TTG​CTG​TAG​CCA​A
PIK3R1 forward	CGC​CTC​TTC​TTA​TCA​AGC​TCG​TG
PIK3R1 reverse	GAA​GCT​GTC​GTA​ATT​CTG​CCA​GG
DDX58 forward	CAC​CTC​AGT​TGC​TGA​TGA​AGG​C
DDX58 reverse	GTC​AGA​AGG​AAG​CAC​TTG​CTA​CC
BIRC6 forward	CAG​CAG​CTC​TTA​TCA​GCA​TGT
BIRC6 reverse	AAC​TGT​GGC​CCA​CTT​AGC​AAC
IFIH1 forward	GCT​GAA​GTA​GGA​GTC​AAA​GCC​C
IFIH1 reverse	CCA​CTG​TGG​TAG​CGA​TAA​GCA​G

### 3.13 Statistical analysis

Statistical analyses were conducted using GraphPad Prism. Quantitative data are presented as mean ± standard deviation. Normality was assessed using the Shapiro-Wilk test, and equality of variances was assessed using Levene’s test. For normally distributed data with homogeneous variance, multiple-group comparisons were performed using one-way analysis of variance followed by an appropriate post hoc multiple-comparison test. For two-group comparisons in cell experiments, an unpaired two-tailed Student’s *t*-test was used when assumptions were met; otherwise, a nonparametric test was applied.

## 4 Results

### 4.1 Serum PON1 levels after CAVD induction, cyanate treatment, and gly intervention

The mouse model’s HDL-associated antioxidant status was assessed using serum PON1. Blank controls were C57BL/6 mice provided a regular diet. The CAVD model group consisted of ApoE−/− mice fed a high-fat diet. The cyanate group consisted of ApoE−/− mice fed a high-fat diet with water containing cyanate, while the inhibitor group consisted of ApoE−/− mice given cyanate plus Gly.

**Figure 1:**
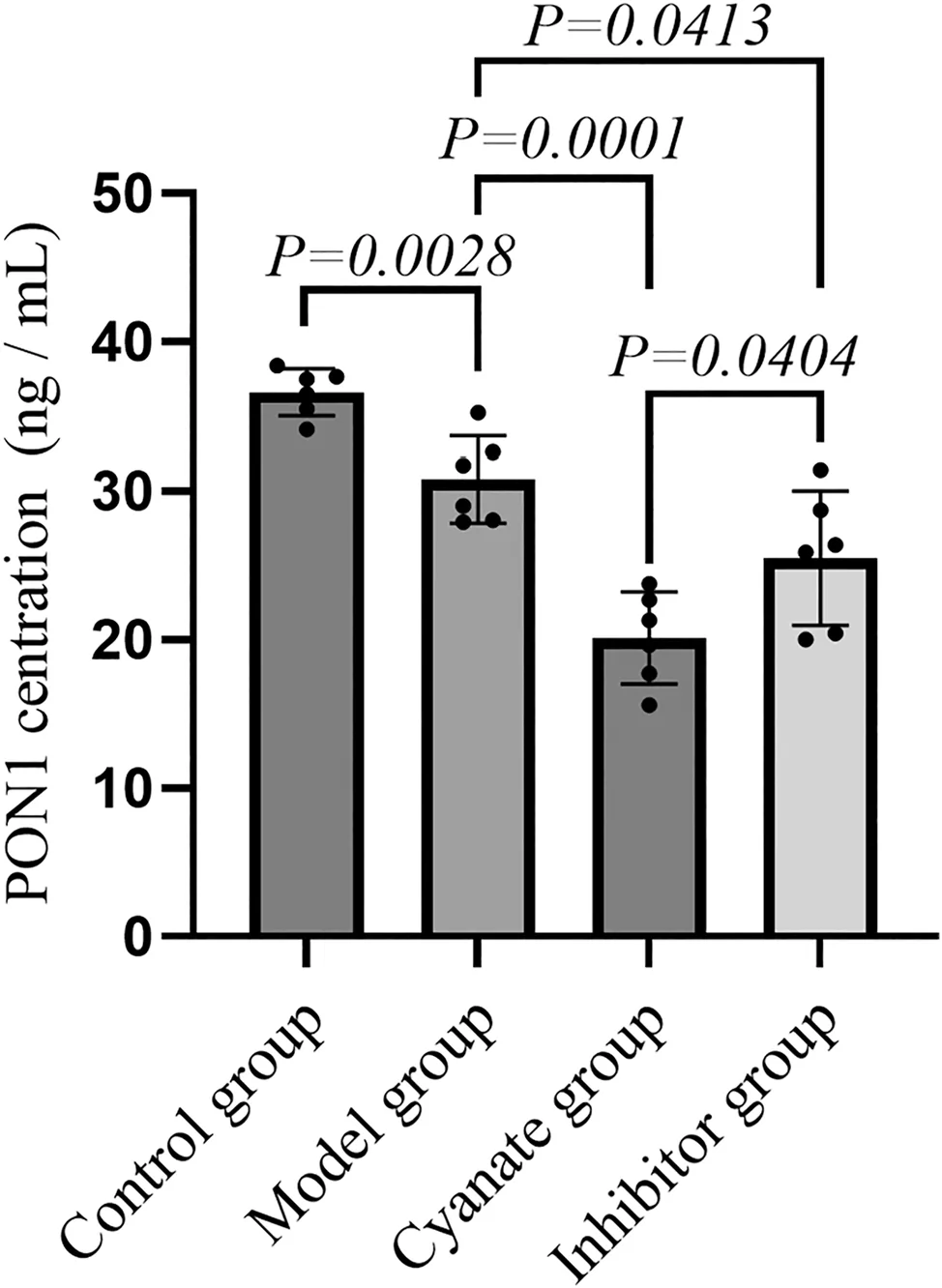
Serum PON1 concentrations in mice from different experimental groups. Bars represent mean ± SD.

The model group’s serum PON1 concentration was lower than that of the blank controls (*P* = 0.0028). The cyanate group’s PON1 was further lower than that of the model group (*P* = 0.0001), while the inhibitor group’s PON1 was higher than that of the cyanate treatment alone (*P* = 0.0404). The inhibitor group’s PON1 levels were considerably lower than those of the model group (*P* = 0.0413) (Figure 1). These results suggest that during CAVD simulation, cyanate intake is linked to a decline in HDL-related antioxidant status, which Gly partially reverses.

### 4.2 Von Kossa staining of aortic valve calcium deposition

**Figure 2:**
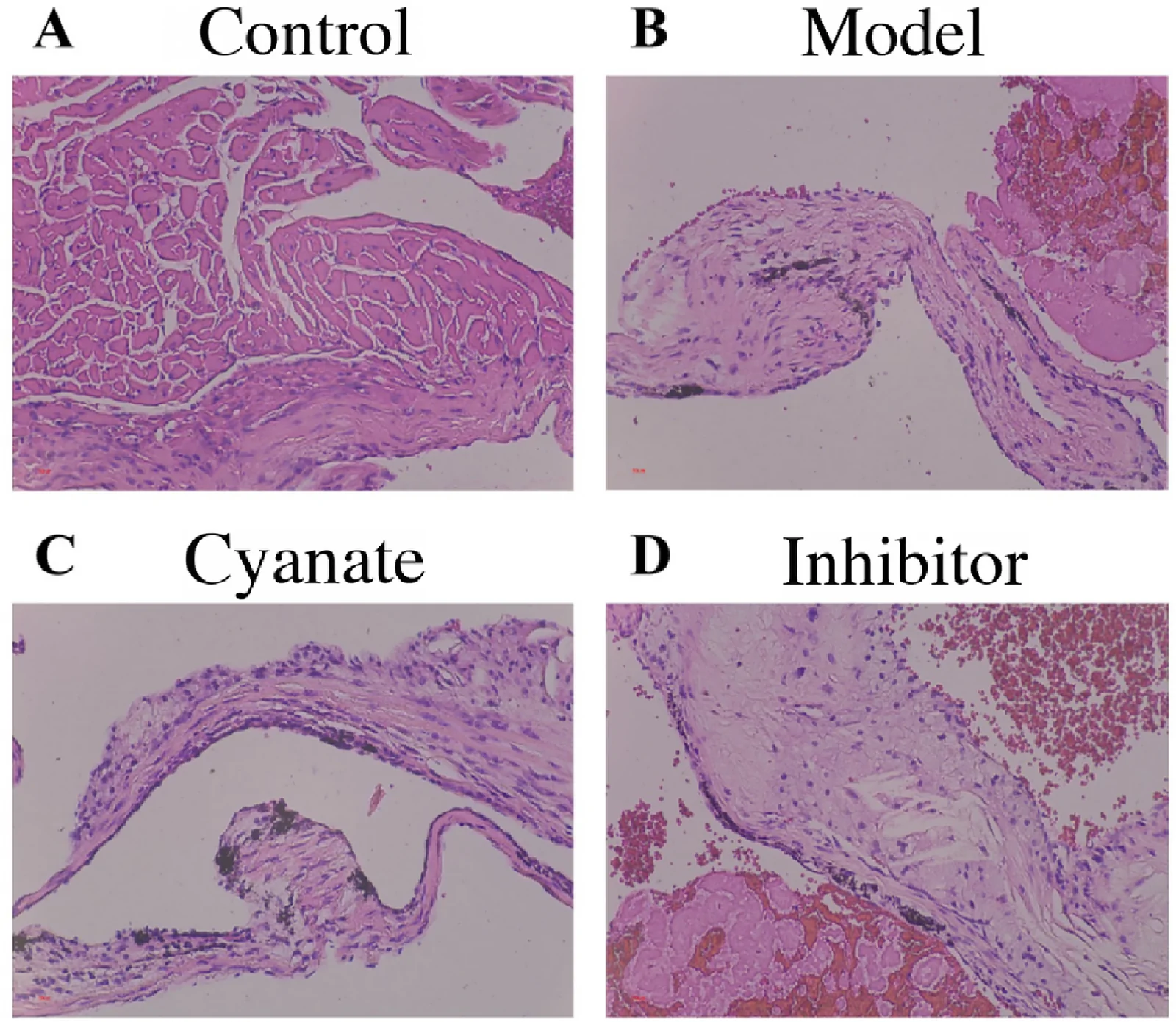
Representative von Kossa staining of aortic valve calcium deposition. Von Kossa staining of aortic valve sections from the control group mice (A), model group mice (B), cyanate group mice (C), and cyanate plus gly inhibitor group mice (D) are shown. Dark deposits indicate calcium-positive regions.

To see the calcium accumulation in each group, aortic root slices with valve leaflets were stained with von Kossa. Blank controls showed no discernible von Kossa-positive staining. While the cyanate group displayed broader, more continuous, or patchy positive regions, the model group displayed localized calcium deposition. When compared to cyanate treatment alone, gly intervention decreased von Kossa-positive staining (Figure 2). These results corroborate a link between increased valve calcification and cyanate-induced carbamylation load.

### 4.3 CD31 immunohistochemistry in aortic valve tissue

**Figure 3:**
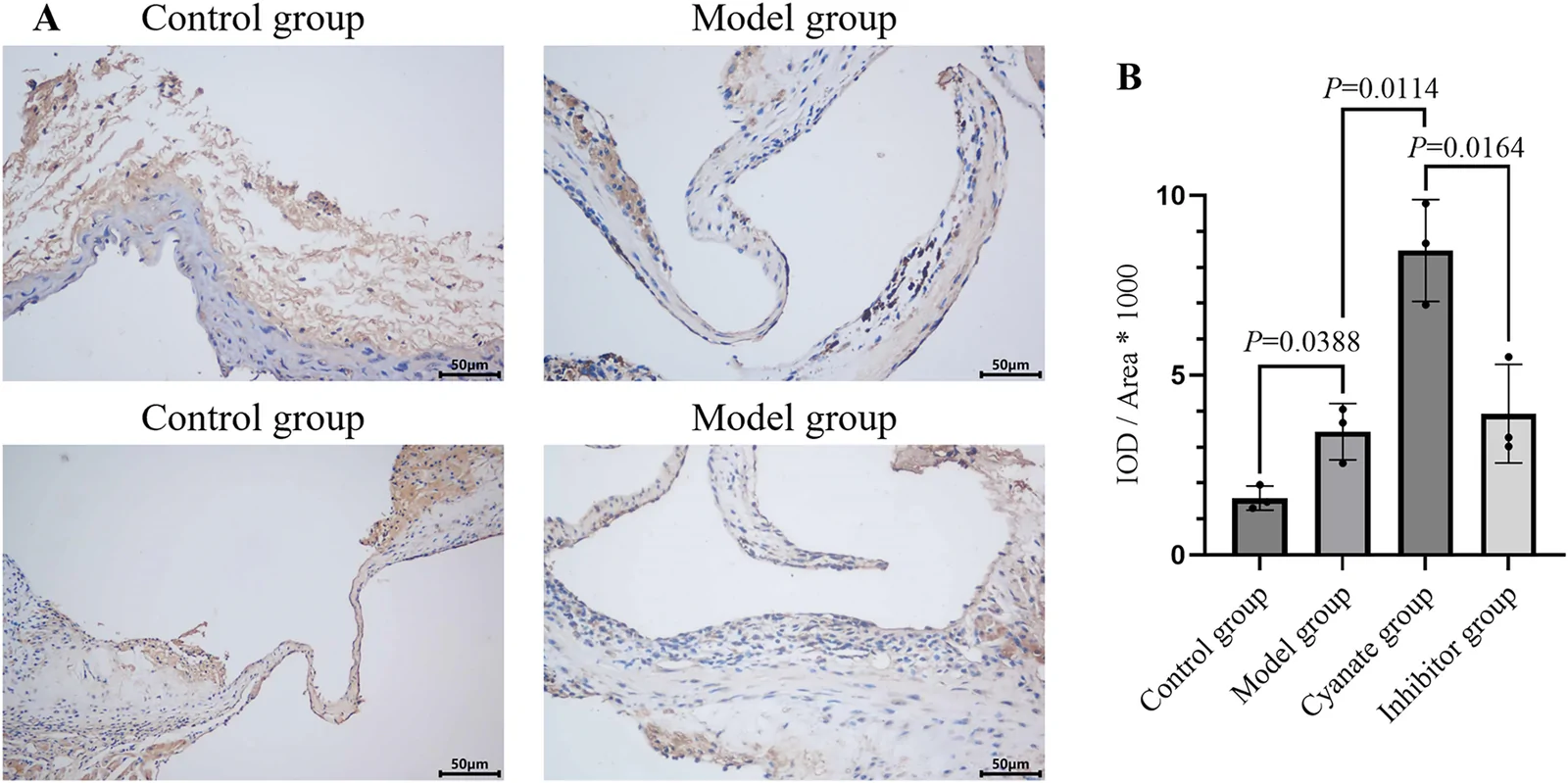
CD31 immunohistochemistry in mice from different experimental groups. (A) Images of CD31 immunohistochemical staining of aortic valve from mice in the control group, model group, cyanate, and inhibitor groups. (B) Quantitative analysis of CD31 immunohistochemical staining of the aortic valve in mice from different groups. Bars represent mean ± SD with individual animal data points.

CD31 immunohistochemistry was utilized to determine endothelial distribution and status alterations in aortic valve tissue. CD31-positive staining was mostly limited to the valvular endothelial layer. In comparison to the control group, the model group had a larger CD31-positive region (*P* = 0.00388). CD31 staining rose more following cyanate treatment than in the model group (*P* = 0.00114), although Gly intervention reduced CD31 positive when compared to cyanate therapy alone (*P* = 0.0164) (Figure 3). Because CD31 is an endothelial marker rather than a particular damage sign, these findings are interpreted as indicating altered endothelial state in the calcification microenvironment.

### 4.4 Expression of p-p65, NOTCH1, and RUNX2 in aortic valve tissue

**Figure 4:**
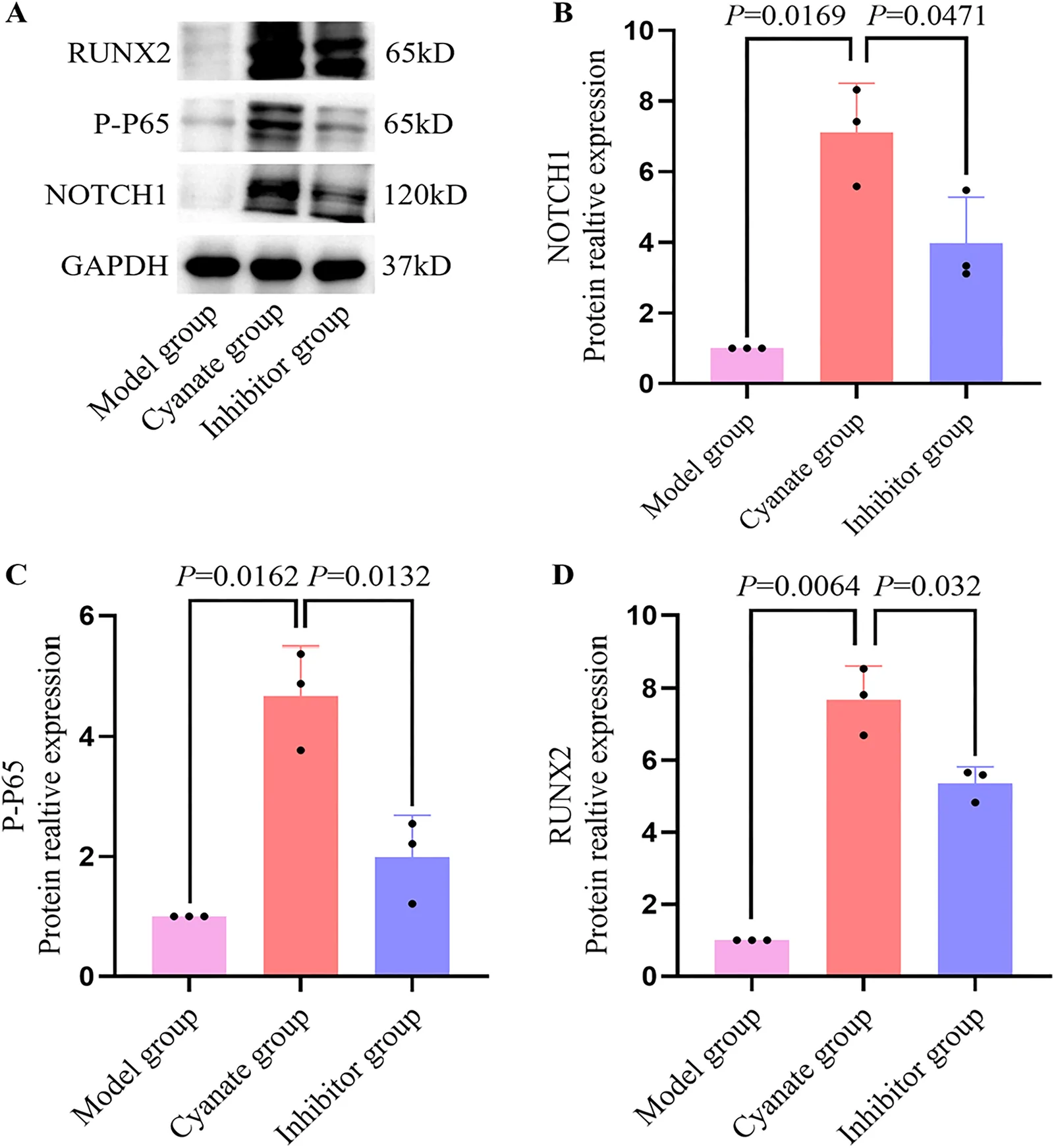
The expressions of P-P65, NOTCH1, and RUNX2 proteins in the aortic valves of mice in different groups. (A) Western blot images of P-P65, NOTCH1, and RUNX2 in the aortic valves of mice in different groups. (B–D) Quantitative analysis of NOTCH1 (B), P-P65 (C) and RUNX2 (D) proteins. Bars represent mean ± SD with individual animal data points.

Western blotting was used to detect inflammatory and osteogenic signaling markers in aortic valve tissue. Compared to the model group, cyanate administration raised p-p65, NOTCH1, and RUNX2 protein levels in valve tissue (*P* < 0.05). Compared to the cyanate group, Gly intervention reduced these protein levels (*P* < 0.05) (Figure 4). Upregulation of p-p65 and RUNX2 indicates activation of the inflammation-osteogenesis signaling pathway, but altered NOTCH1 expression shows involvement in valve developmental and homeostatic signaling.

### 4.5 RNA-seq identification of C-HDL-associated DEGs in AVICs

**Figure 5:**
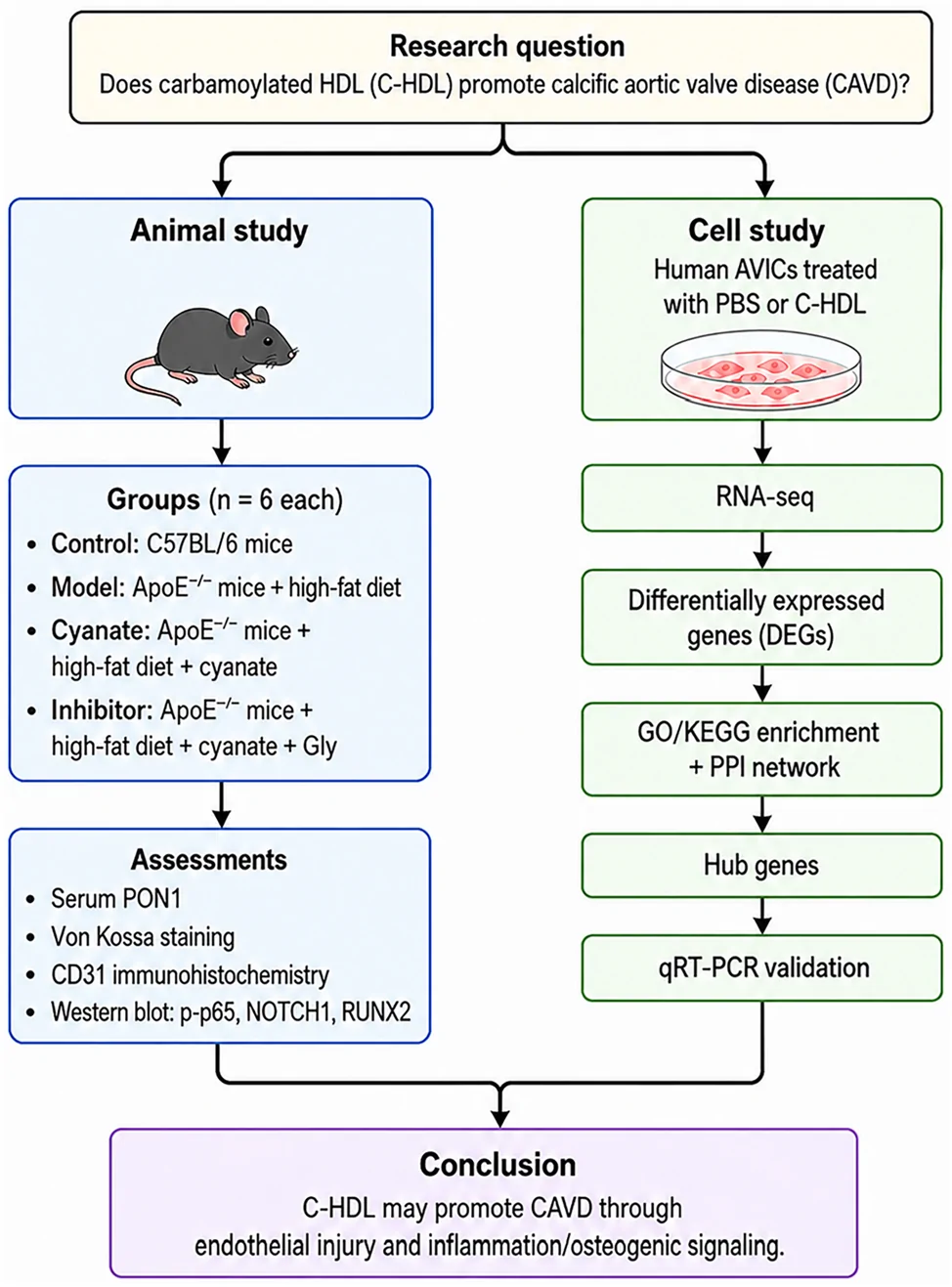
Study workflow for animal experiments, RNA-seq analysis, enrichment analysis, PPI-network construction, and qRT-PCR validation.

**Figure 6:**
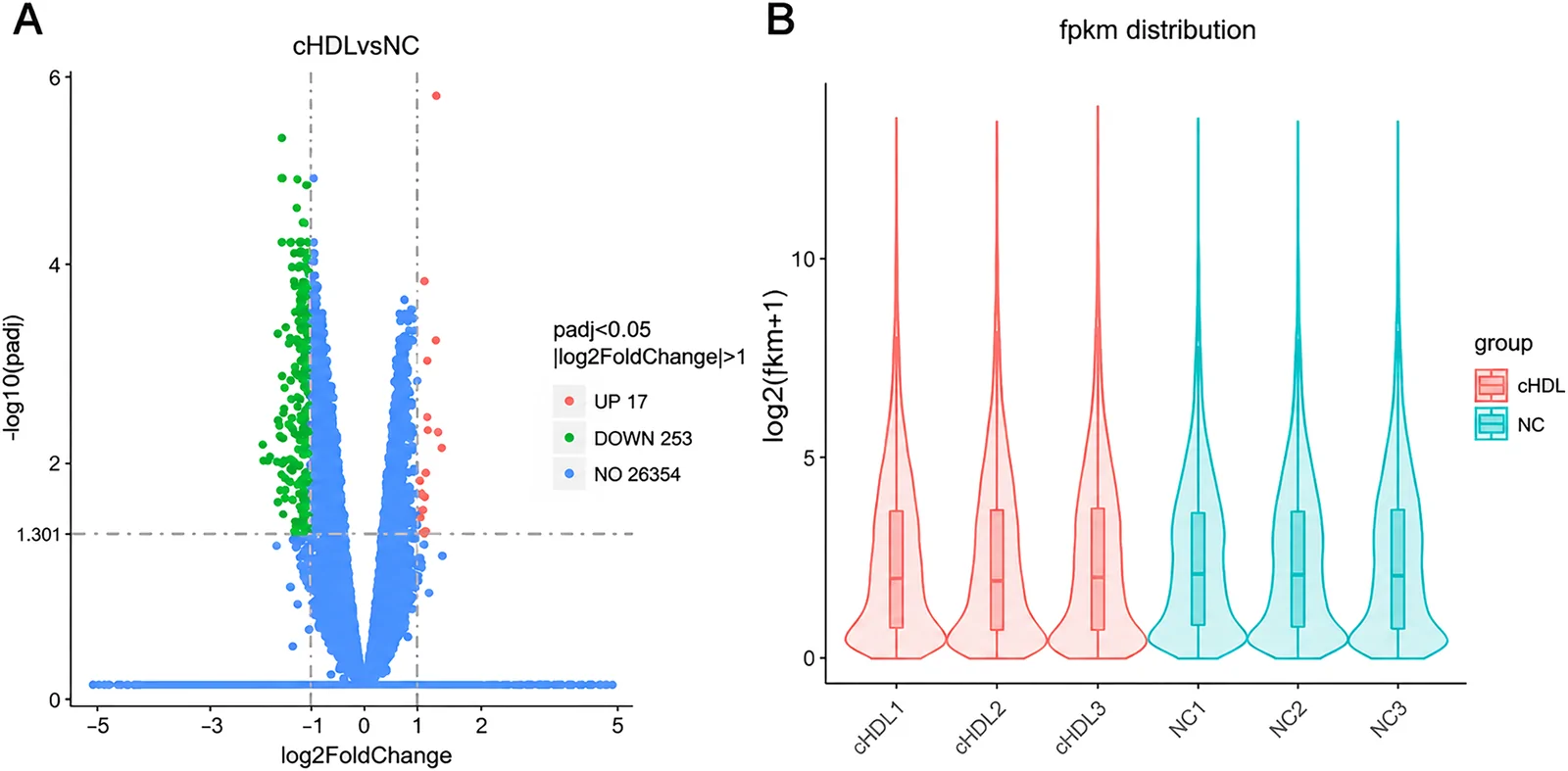
Normalized expression distribution and volcano plot of C-HDL-associated DEGs in AVICs.

Human AVICs were treated with either PBS or C-HDL (75 μg/mL) to ascertain the direct transcriptional response of valve interstitial cells to C-HDL. RNA was extracted and put through RNA-seq after 8 h (Figure 5). To assess sample comparability, normalized expression distributions were examined. 270 DEGs, comprising 253 downregulated and 17 upregulated genes, were found using absolute log2 fold change ≥1 and adjusted *P* < 0.05 as thresholds (Figure 6).

### 4.6 GO and KEGG enrichment results for C-HDL-associated DEGs

**Figure 7:**
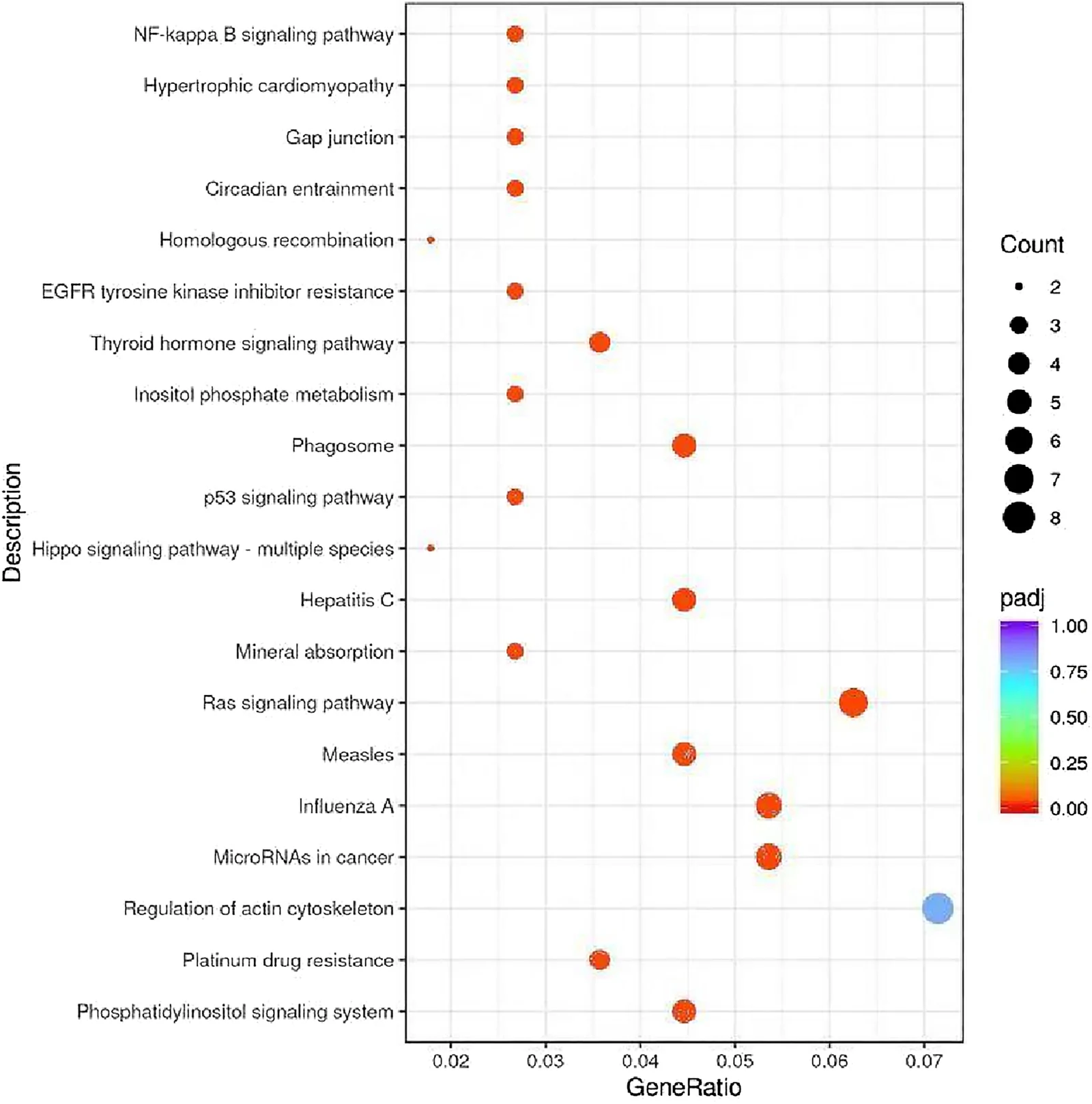
KEGG enrichment analysis of C-HDL-associated DEGs.

**Figure 8:**
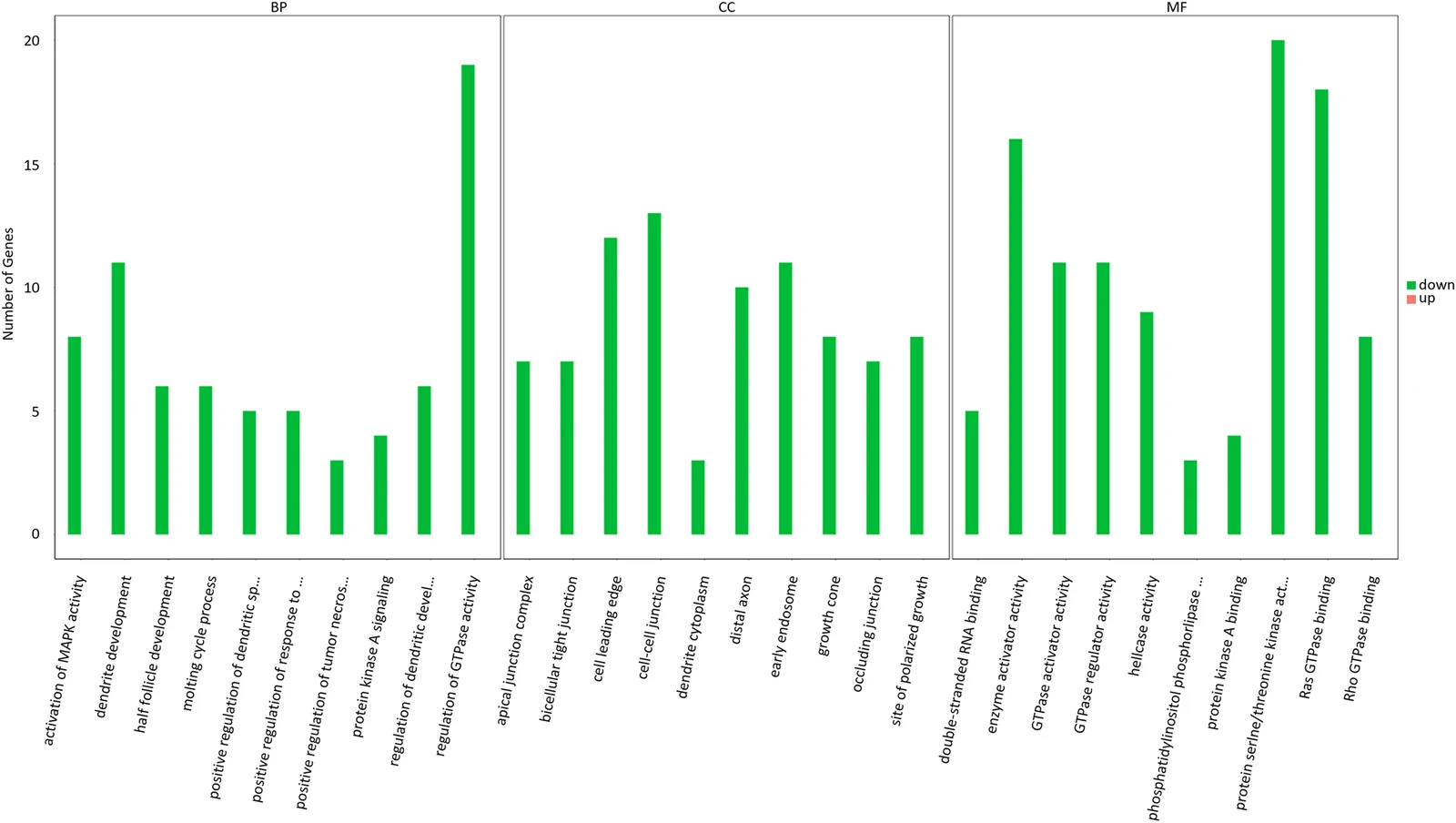
GO enrichment analysis of C-HDL-associated DEGs.

C-HDL-associated DEGs have been demonstrated to be abundant in pathways such as NF-κB signaling, gap junction, actin cytoskeleton regulation, phosphatidylinositol signaling, and cardiovascular/stress-related pathways, according to KEGG analysis (Figure 7). GO analysis revealed enrichment in biological processes and functions associated with cell signaling, cell-cycle control, cell-cell junctions, apical functional complexes, GTPase regulatory activity, and protein kinase A binding (Figure 8). Together, these findings indicate that C-HDL influences transcriptional programs involved in intercellular communication, cytoskeletal architecture, inflammatory signaling, and valve-tissue integrity.

### 4.7 PPI network and hub-gene screening

**Figure 9:**
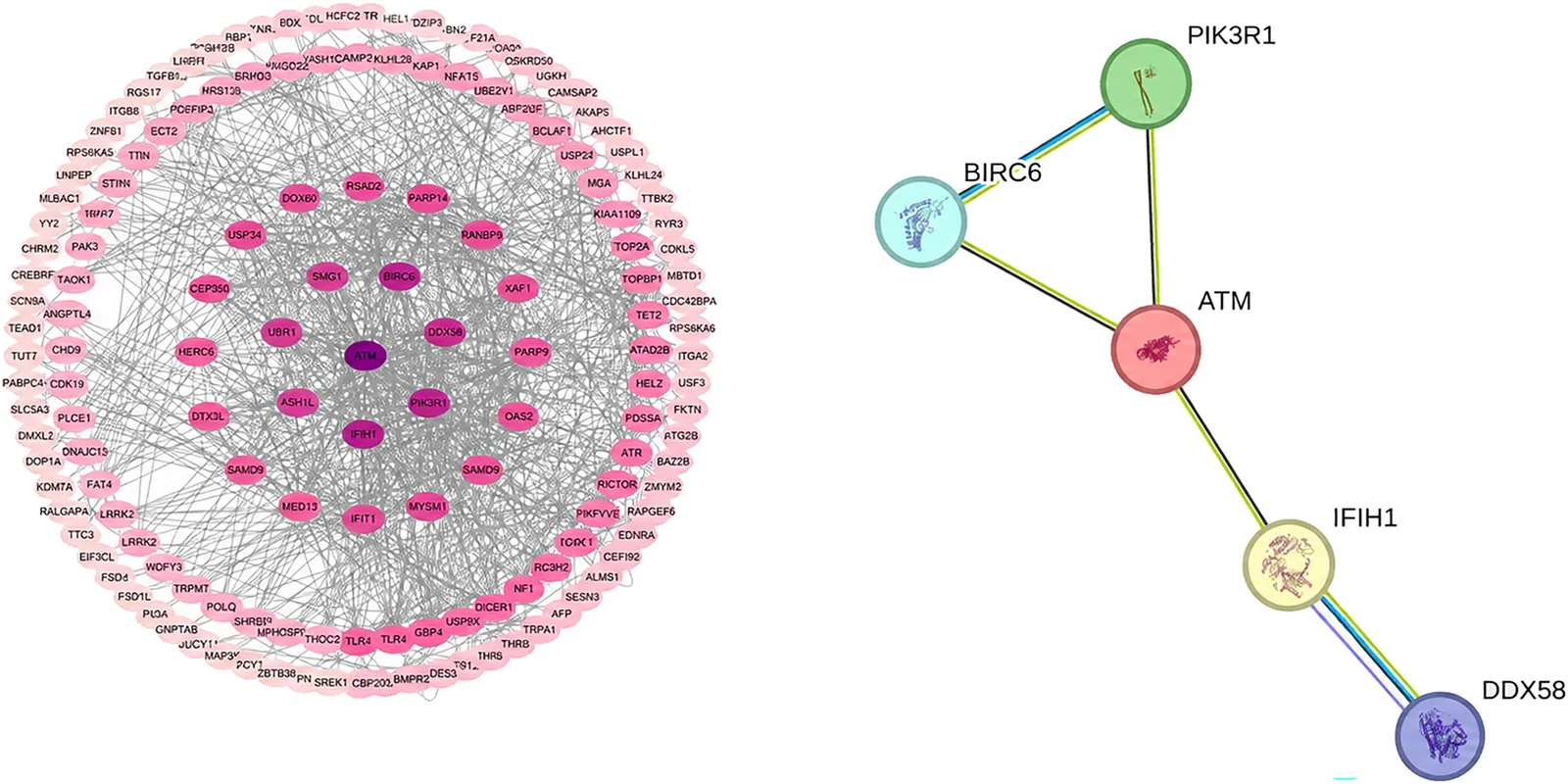
PPI-network analysis of C-HDL-associated DEGs and hub-gene selection.

To find strongly linked genes among the C-HDL-associated DEGs, a PPI network was created using STRING and displayed in Cytoscape. 153 interaction nodes were kept when the combined score criterion was greater than 0.4. Five hub genes were found for further validation using topological analysis and the Molecular Complex Detection module: BIRC6, PIK3R1, ATM, IFIH1, and DDX58 (Figure 9).

### 4.8 qRT-PCR validation of candidate hub genes in AVICs

**Figure 10:**
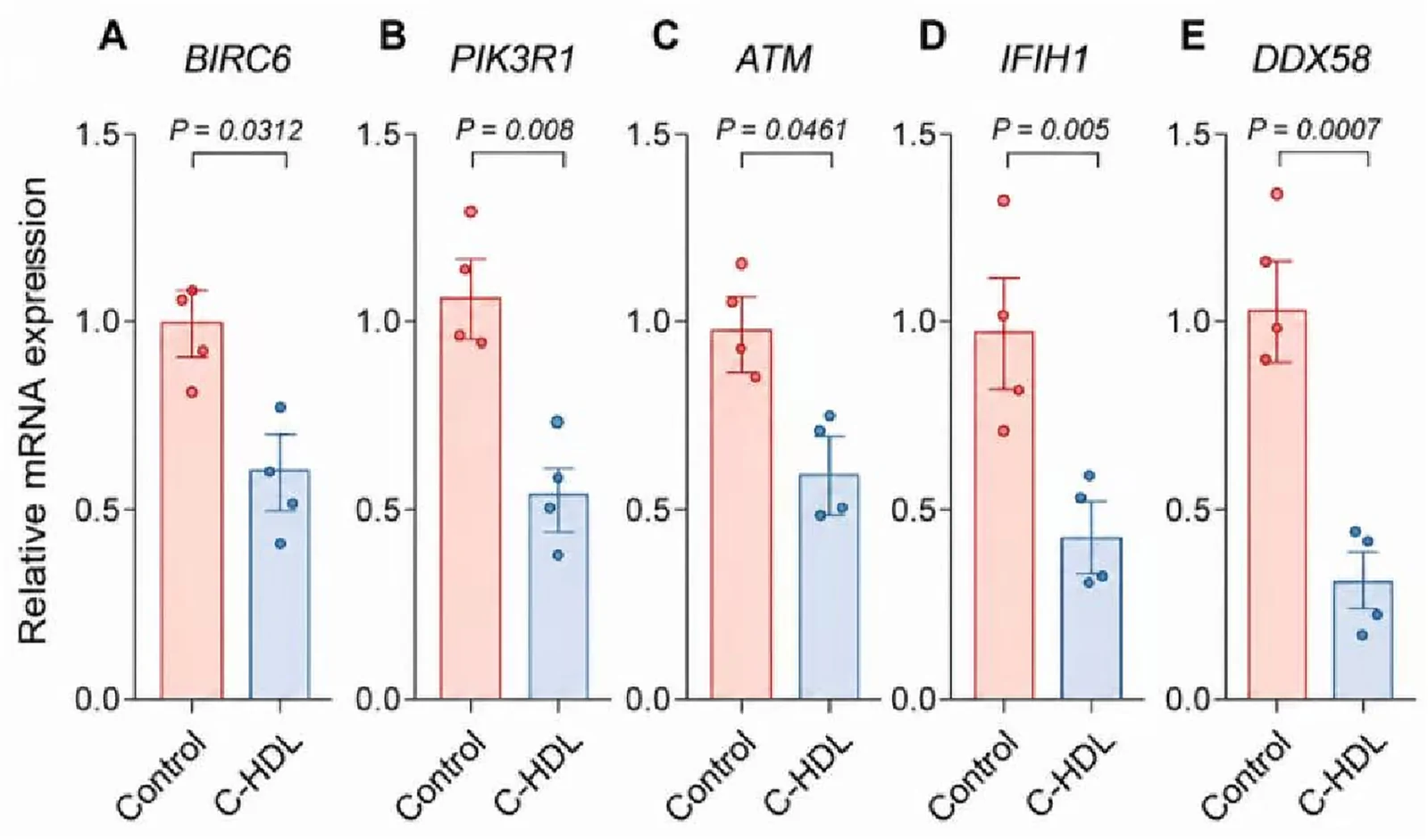
qRT-PCR validation of BIRC6, PIK3R1, ATM, IFIH1, and DDX58 expression in AVICs. Bars represent mean ± SD with individual biological data points. Exact *P* values should be provided in the figure source-data file.

AVICs were cultured under calcification-inducing conditions and treated with either PBS or C-HDL in order to validate RNA-seq results. In accordance with the direction of change found by RNA-seq, qRT-PCR revealed that BIRC6, PIK3R1, ATM, IFIH1, and DDX58 mRNA levels were lower in the C-HDL group than in controls (Figure 10). These genes could be potential nodes that connect C-HDL activation to innate immunological signaling, cell-survival control, stress responses, and remodeling associated with calcification.

## 5 Discussion

CAVD is a fibrocalcific disorder that triggers lipoprotein dysfunction, endothelial disruption, inflammatory activation, extracellular matrix remodeling, and osteogenic differentiation of valvular stromal cells [2], 3], 13]. The current study investigated C-HDL as a potential contributor to these processes using an *in vivo* cyanate-associated carbamylation model and AVIC-based transcriptome analysis. Our data indicates that cyanate exposure is linked to decreased serum PON1, increased valve calcium deposition, altered CD31 staining, and overexpression of p-p65, NOTCH1, and RUNX2. Gly administration reduced these alterations, indicating a possible relationship between carbamylation-related lipoprotein dysfunction and CAVD development.

HDL typically has antioxidant, anti-inflammatory, and endothelial-protective properties; nevertheless, HDL can become dysfunctional in inflammatory, metabolic, and chronic renal disease conditions [6], [7], [8], [9]. PON1 is an HDL-associated antioxidant enzyme that helps to maintain HDL’s protective activity. Carbamylation is a significant post-translational modification of proteins that occurs *in vivo*, potentially resulting in alterations to protein structure and function. In this work, serum PON1 levels were reduced in mice with CAVD-related alterations and decreased even more after cyanate treatment. These findings indicate that carbamylated high-density lipoprotein may contribute to CAVD development by inhibiting the function of HDL-associated antioxidant enzymes.

Previous research has connected carbamylated lipoproteins to endothelial dysfunction, inflammation, and unfavorable cardiovascular remodeling [11]. This provides experimental evidence that cyanate-related stimuli affect valvular endothelial homeostasis, supporting the hypothesis that cyanate treatment may induce endothelial stress and functional impairment. The increased CD31-positive area found following cyanate administration could imply changes in endothelial distribution, junctional state, or inflammation-induced endothelial remodeling. When combined with increased calcification and altered inflammatory-osteogenic signaling, the CD31 data support the idea that cyanate-related stimuli disturb valve endothelial homeostasis during CAVD progression.

NF-κB is a crucial inflammatory pathway that can interact with osteogenic transcriptional programs at the signaling level. Post-translational modulation of p65 is increasingly acknowledged as a key mechanism regulating the intensity and duration of inflammatory signaling, and phosphorylation of p65 is frequently utilized as a marker of NF-κB activation [16]. Our previous studies indicate that C-HDL may play a role in the calcification process of aortic valve stromal cells by activating TLR4. This activation can influence signaling pathways such as NOTCH1 and NF-κB. Inhibiting these pathways can help reduce the calcification effects induced by C-HDL. Building on this background, the current study further investigated changes in the expression of relevant proteins in aortic valve tissue. In this work, Gly partially restored the effects of cyanate therapy on p-p65 and RUNX2 expression in valve tissue. Additionally, NOTCH1 expression rose following cyanate exposure and feel following Gly intervention. These results align with a concept of valve calcification based on inflammation and osteogenesis.

Following C-HDL treatment of AVICs, 270 DEGs were found by transcriptome analysis. NF-κB-related pathways, gap junctions, cytoskeletal control, phosphatidylinositol signaling, cell-cell junctions, and GTPase regulatory activities were all implicated, according to functional enrichment. Importantly, the activation of NF-κB-related pathways highlights that inflammatory signaling is likely a crucial molecular mechanism driving C-HDL-associated aortic valve calcification. These findings are consistent with previous discussions regarding the role of inflammatory responses in CAVD and the potential involvement of NF-κB signaling in the calcification process. These results suggest that exposure to C-HDL may impact tissue integrity, inflammatory signaling, cytoskeletal architecture, and cellular communication. BIRC6, PIK3R1, ATM, IFIH1, and DDX58 were also found to be potential hub genes by PPI-network analysis. These genes, which may be connected to stress reactions, innate immunological signaling, DNA-damage responses, cell survival, and remodeling related to calcification, were downregulated in the C-HDL group. Our findings align with previous studies indicating that C-HDL-induced transcriptional changes are closely associated with the inflammatory response and extracellular matrix remodeling in valvular interstitial cells, core biological processes that drive aortic valve calcification. The hub genes identified through PPI network analysis in this study are mainly enriched in pathways closely related to cell proliferation and inflammatory activation, thereby complementing the existing understanding of the transcriptional mechanisms underlying C-HDL-mediated aortic valve calcification.

The importance of machine learning, deep learning, feature selection, and network-based techniques for finding disease-related molecular markers and possible treatment candidates has been highlighted more and more by recent developments in computational biology. For example, feature-selection or feature-reconstruction techniques have improved biological classification tasks, such as organelle protein classification and image-derived disease classification; attention-based deep autoencoder frameworks have improved clustering of high-dimensional single-cell RNA-seq data; and capsule network-based models have been applied to disease-related compound identification [17], [18], [19], [20]. These studies demonstrate how crucial computational methods are for obtaining physiologically significant data from intricate biomedical datasets. In keeping with this trend in methodology, the current work incorporated RNA-seq,GO/KEGG enrichment analysis, PPI-network creation, and hub-gene screening are used to investigate C-HDL-associated transcriptional alterations in AVICs. Unlike solely predictive computational models, our analysis included both *in vivo* phenotypic assessment and qRT-PCR validation, giving preliminary biological evidence for the identified C-HDL-associated molecular markers. These findings remain exploratory, and further functional experiments are required to clarify the causal roles of the identified hub genes in C-HDL-induced valvular inflammation and osteogenic differentiation.

However, this study still has some limitations. First, neither PON1 enzyme activity nor HDL carbamylation were measured. Second, endothelial activation or damage cannot be determined only by CD31 expression. Third, only total NOTCH1, p-p65, and RUNX2 were included in the Western blot results; further pathway markers and functional inhibition studies are required. Fourth, RNA-seq and network analysis were used to identify the potential hub genes, which need to be mechanistically validated. Lastly, generalizability is constrained by the comparatively small animal sample size. Direct assessment of HDL carbamylation, PON1 activity assays, pathway-intervention studies, bigger animal and clinical valve-tissue cohorts, and multi-omics integration should all be included in future research.

## 6 Conclusions

C-HDL may contribute to CAVD progression by altering valvular endothelial homeostasis and activating inflammation-osteogenesis signaling, as evidenced by elevated p-p65, NOTCH1, and RUNX2 expression. Gly intervention partially corrected cyanate-mediated biochemical, histological, endothelial, and signaling alterations. Transcriptomic and PPI-network analysis also identified BIRC6, PIK3R1, ATM, IFIH1, and DDX58 as possible hub genes in C-HDL-associated CAVD. These data lend support to the idea that C-HDL-related lipoprotein dysfunction may be a contributing factor to valve calcification.

## Supplementary Material

Supplementary Material

Supplementary Material
