# Supplementary material for: Mechanisms by which carbamoylated high-density lipoprotein (C-HDL) promotes calcific aortic valve disease and exploration of potential targeted therapies
Source: Open Life Sci. 2026 Sep 8;21(1):20251367. doi: 10.1515/biol-2025-1367 (PMC13552616; doi:10.1515/biol-2025-1367)
Supplement: Supplementary file 1 [file j_biol-2025-1367_suppl_001.docx]

suppressPackageStartupMessages({
library(edgeR)
library(limma)
library(dplyr)
library(readr)
library(tibble)
library(ggplot2)
library(pheatmap)


Set working directory
setwd("D:/RNAseq_CHDL_AVICs/")

Read input files
count_raw <- read.csv("raw_counts.csv", check.names = FALSE)
sample_info <- read.csv("sample_info.csv", check.names = FALSE)

Basic checking
stopifnot("gene_id" %in% colnames(count_raw))
stopifnot("sample" %in% colnames(sample_info))
stopifnot("group" %in% colnames(sample_info))has_symbol <- "gene_symbol" %in% colnames(count_raw)

Clean gene IDs
count_raw$gene_id <- gsub("\\..*$", "", count_raw$gene_id)

Extract count matrix
gene_anno_cols <- c("gene_id", "gene_symbol")
gene_anno_cols <- gene_anno_cols[gene_anno_cols %in% colnames(count_raw)]

expr_cols <- setdiff(colnames(count_raw), gene_anno_cols)

保证样本顺序与 sample_info 一致
expr_cols <- sample_info$sample
stopifnot(all(expr_cols %in% colnames(count_raw)))

count_mat <- count_raw[, expr_cols]
rownames(count_mat) <- count_raw$gene_id

 Convert to numeric matrix
count_mat <- as.data.frame(count_mat)
count_mat[] <- lapply(count_mat, function(x) as.numeric(as.character(x)))
count_mat <- as.matrix(count_mat)

Remove genes with missing values
na_gene_number <- sum(!complete.cases(count_mat))
message("Genes with missing values removed: ", na_gene_number)

count_mat <- count_mat[complete.cases(count_mat), ]

 Remove genes with negative counts
raw counts 不应出现负值
negative_gene_number <- sum(rowSums(count_mat < 0) > 0)
message("Genes with negative values removed: ", negative_gene_number)

count_mat <- count_mat[rowSums(count_mat < 0) == 0, ]

 Remove duplicated gene IDs
count_df <- as.data.frame(count_mat)
count_df$gene_id <- rownames(count_mat)

count_df <- count_df %>%
 mutate(total_count = rowSums(across(all_of(expr_cols)))) %>%
 arrange(gene_id, desc(total_count)) %>%
 distinct(gene_id, .keep_all = TRUE) %>%
 select(-total_count)

rownames(count_df) <- count_df$gene_id
count_df$gene_id <- NULL
count_mat <- as.matrix(count_df)

message("Genes retained after duplicate removal: ", nrow(count_mat))

 Remove genes with all-zero counts
all_zero_gene_number <- sum(rowSums(count_mat) == 0)
message("All-zero genes removed: ", all_zero_gene_number)

count_mat <- count_mat[rowSums(count_mat) > 0, ]

Build DGEList object
sample_info$group <- factor(sample_info$group, levels = c("Control", "C_HDL"))

dge <- DGEList(
 counts = count_mat,
 group = sample_info$group
)

Filter lowly expressed genes
 edgeR::filterByExpr
keep <- filterByExpr(dge, group = sample_info$group)

message("Genes before low-expression filtering: ", nrow(dge))
message("Genes after low-expression filtering: ", sum(keep))

dge <- dge[keep, , keep.lib.sizes = FALSE]

 Library size normalization
dge <- calcNormFactors(dge, method = "TMM")

 Generate logCPM matrix for QC and visualization
logCPM <- cpm(dge, log = TRUE, prior.count = 1)

Save cleaned matrices
write.csv(
 dge$counts,
 file = "cleaned_raw_counts_filtered.csv",
 quote = FALSE
)

write.csv(
 logCPM,
 file = "cleaned_logCPM_TMM_normalized.csv",
 quote = FALSE
)

write.csv(
 sample_info,
 file = "cleaned_sample_info.csv",
 row.names = FALSE,
 quote = FALSE
)

Sample library size summary
lib_summary <- data.frame(
 sample = colnames(dge$counts),
 group = sample_info$group,
 raw_library_size = colSums(count_mat[, colnames(dge$counts)]),
 filtered_library_size = colSums(dge$counts),
 norm_factor = dge$samples$norm.factors
)

write.csv(
 lib_summary,
 file = "sample_library_size_summary.csv",
 row.names = FALSE,
 quote = FALSE
)

Boxplot of logCPM distribution
pdf("QC_boxplot_logCPM.pdf", width = 7, height = 5)
boxplot(
 logCPM,
 las = 2,
 main = "Distribution of TMM-normalized logCPM values",
 ylab = "log2 CPM"
)
dev.off()


write.csv(
 pca_df,
 file = "QC_PCA_coordinates.csv",
 row.names = FALSE,
 quote = FALSE
)

Sample correlation heatmap
sample_cor <- cor(logCPM, method = "pearson")

pdf("QC_sample_correlation_heatmap.pdf", width = 6, height = 5)
pheatmap(
 sample_cor,
 main = "Sample correlation heatmap",
 clustering_distance_rows = "correlation",
 clustering_distance_cols = "correlation"
)
dev.off()

write.csv(
 sample_cor,
 file = "QC_sample_correlation_matrix.csv",
 quote = FALSE
)

Optional: remove obvious outlier samples manually
 outlier_samples <- c("NC_3")
 dge <- dge[, !colnames(dge) %in% outlier_samples]
 sample_info <- sample_info[!sample_info$sample %in% outlier_samples, ]

message("Expression matrix cleaning completed successfully.")

Supplementary Code 02
 Differential expression analysis using limma-voom

suppressPackageStartupMessages({
 library(edgeR)
 library(limma)
 library(ggplot2)
 library(dplyr)
})


 Contrast: C-HDL vs Control
contrast_matrix <- makeContrasts(
 C_HDL_vs_Control = C_HDL - Control,
 levels = design
)

fit2 <- contrasts.fit(fit, contrast_matrix)
fit2 <- eBayes(fit2)

 Export all DEG results
deg_all <- topTable(
 fit2,
 coef = "C_HDL_vs_Control",
 number = Inf,
 adjust.method = "BH"
)

deg_all$gene_id <- rownames(deg_all)

write.csv(
 deg_all,
 file = "DEG_all_limma_voom_C_HDL_vs_Control.csv",
 row.names = FALSE,
 quote = FALSE
)

Set DEG threshold
deg_all <- deg_all %>%
 mutate(
 regulation = case_when(
 adj.P.Val < 0.05 & logFC >= 1 ~ "Upregulated",
 adj.P.Val < 0.05 & logFC <= -1 ~ "Downregulated",
 TRUE ~ "Not significant"
 )
 )

deg_sig <- deg_all %>%
 filter(adj.P.Val < 0.05, abs(logFC) >= 1)

write.csv(
 deg_sig,
 file = "DEG_significant_adjP0.05_logFC1.csv",
 row.names = FALSE,
 quote = FALSE
)

message("Number of significant DEGs: ", nrow(deg_sig))
message("Upregulated genes: ", sum(deg_sig$logFC >= 1))
message("Downregulated genes: ", sum(deg_sig$logFC <= -1))

 Volcano plot
pdf("volcano_plot_C_HDL_vs_Control.pdf", width = 6, height = 5)
ggplot(deg_all, aes(x = logFC, y = -log10(adj.P.Val), color = regulation)) +
 geom_point(alpha = 0.7, size = 1.5) +
 theme_bw() +
 geom_vline(xintercept = c(-1, 1), linetype = "dashed") +
 geom_hline(yintercept = -log10(0.05), linetype = "dashed") +
 labs(
 title = "Differentially expressed genes: C-HDL vs Control",
 x = "log2 fold change",
 y = "-log10 adjusted P value"
 )
dev.off()
